# A severe early presentation of cystic fibrosis in an infant with a homozygous c.1375_1383del CFTR variant- a case report

**DOI:** 10.3389/fped.2025.1643050

**Published:** 2025-08-26

**Authors:** Abdullah Yousef

**Affiliations:** ^1^ College of Medicine, Imam Abdulrahman Bin Faisal University, Dammam, Saudi Arabia; ^2^ Department of Pediatrics, King Fahd Hospital of the University, Imam Abdulrahman Bin Faisal University, Al Khobar, Saudi Arabia

**Keywords:** cystic fibrosis, genes, mutation, infections, CFTR protein

## Abstract

**Background:**

Cystic fibrosis is a genetic disease affecting mainly the respiratory and digestive systems through CFTR gene mutations. The condition is characterized by the production of thick mucus, which can lead to severe respiratory complications and pancreatic insufficiency.

We report a rare homozygous c.1375_1383del CFTR variant associated with early, clinically significant presentation. This highlights the importance of early recognition and genotype-specific management to maximize patient outcomes and improve quality of life.

**Case Report:**

We present a 10-month-old female infant born to consanguineous parents with a significant medical history of chronic cough, cyanosis, failure to thrive, poor feeding, and irritability who ultimately required multiple hospitalizations for severe infections requiring mechanical ventilation and intravenous antibiotics. Initial evaluations included thorough clinical assessments and several diagnostic tests, including whole-exome sequencing, which revealed a homozygous c.1375_1383del variant in the CFTR gene. Aggressive therapy, including antipseudomonal antibiotics, was needed to clear the infection, in addition to administration of dornase alpha, 7% hypertonic saline, and pancreatic enzyme replacement therapy. These interventions contributed significantly to the gradual clinical improvement of the patient. At 18-month follow-up, the patient exhibited improved weight gain and a reduction in the frequency of exacerbations.

**Conclusion:**

The c.1375_1383del variant is a rare CFTR variant and is associated with early, clinically significant manifestations of cystic fibrosis in infants, which necessitates early recognition and aggressive management to improve patient outcomes. This case underscores the need for awareness of rare CFTR variants and their potential clinical implications, which can lead to tailored treatment approaches, ultimately enhancing the quality of life for affected individuals.

## Introduction

Cystic fibrosis (CF) is a genetic disorder affecting various systems in the body, most importantly, the respiratory and digestive systems. It is caused by mutations in the cystic fibrosis transmembrane conductance regulator (CFTR) gene, which disrupts ion transport, resulting in the production of thick secretions that significantly impact the quality of life of affected individuals (1).

The incidence of CF in the Middle East has been reported to be 1:2,500–5,000 (2). More than 2,000 CFTR mutations have been reported, which are classified into 6 main categories according to the defect in the CFTR protein (3). There is significant clinical heterogeneity in CF, with a wide range of symptoms and disease severity. This variability often complicates the diagnosis and management of this disease.

CF was first reported in Saudi Arabia in 1986, when an infant presented with recurrent respiratory infections, diarrhea, and failure to thrive (4). F508del is estimated to constitute approximately 12% of CFTR mutations in Saudi Arabia. The CFTR mutation 1,548 delG is the most common in Saudi Arabia (20%). The most common CFTR mutations of Saudi ethnic origin are 1,548 delG, F508del, I1234V, 3,120 1G > A, H139l, 711 1G > A, N1303K, S549R, 2043delG, and 1507del9, which constitute approximately 80% of the reported mutations (2). The eastern province of Saudi Arabia has the highest reported prevalence of CF, at 1:3,000 (5). This high prevalence necessitates increased awareness and screening in the region. The purpose of this report is to describe an infantile phenotype with significant complications of a patient with cystic fibrosis linked to the rare c.1375_1383del variant and highlight challenges in managing rare CFTR variants in high-consanguinity regions like Saudi Arabia.

## Case report

A 10-month-old female infant, born to first-degree consanguineous parents, presented with a history of progressive chronic cough, episodic cyanosis, progressive feeding difficulty, irritability, and failure to thrive (weight <5th percentile). She was born at term via an uncomplicated delivery with no neonatal complications, including no meconium ileus or jaundice. At six months of age, she experienced her first hospitalization for severe bronchiolitis, requiring 11 days of high-flow oxygen and supportive care. Over the next three months, she had three additional admissions for recurrent respiratory failure; two of which progressed to acute respiratory distress syndrome (ARDS), necessitating pediatric intensive care unit (PICU) admission, mechanical ventilation, and intravenous antibiotics.

Upon feeding resumption, her caregivers noted persistent loose stools, previously masked by prolonged periods of nil per os status. Her family history was notable for parental consanguinity, but no chronic respiratory disorders.

Her examination showed a malnourished infant (weight 5.5 kg < 5th percentile), tachypneic with retractions and cyanosis, due to which she was given oxygen and later required intubation and mechanical ventilation. Her chest was barrel-shaped, and auscultation detected bilateral coarse crepitations with no digital clubbing in her hands.

A blood gas examination revealed evidence of respiratory failure with increased inflammatory markers. Serum electrolytes were normal. Chest computed tomography (CT) demonstrated peribronchial thickening and consolidation with a ground-glass appearance and signs of early bronchiectasis.

The patient underwent flexible bronchoscopy with bronchoalveolar lavage, and cultures identified Pseudomonas aeruginosa, prompting antibiotic treatment. The classical triad of chronic cough, steatorrhea, failure to thrive, alongside recurrent Pseudomonas aeruginosa infections and early bronchiectasis, strongly supported a diagnosis of CF. Whole-exome sequencing (WES) was prioritized to confirm CF and exclude alternative diagnoses such as PCD and immunodeficiency. This approach identified a homozygous rare variant, c.1375_1383del in the CFTR gene, confirming the diagnosis of cystic fibrosis.

Following confirmation of the homozygous c.1375_1383del CFTR variant in the patient, genetic counseling was offered to the parents and siblings to discuss carrier status and implications for future family planning. The family, however, declined further genetic testing at this time.The patient was started on dornase alfa, 7% hypertonic saline with chest physiotherapy, and pancreatic enzyme replacement therapy, after which her weight started to improve. She was administered a prolonged course of antipseudomonal therapy, including intravenous antibiotics and inhaled colistin, guided by sensitivity testing.

Following treatment initiation, the patient experienced a significant reduction in respiratory exacerbations. Before treatment, she required four hospitalizations for severe respiratory exacerbations within three months, two of which progressed to acute respiratory distress syndrome (ARDS). Since starting treatment, over the next 18 months, she has had a few mild pulmonary exacerbations, all managed successfully with oral antibiotic courses in the outpatient setting, with no further hospitalizations required. She was also referred to a CF center and continued outpatient care. She gained weight, reaching the 25th percentile.

The current status of Pseudomonas aeruginosa colonization—whether persistent or intermittent—remains unknown. Ongoing microbiological surveillance at the current care center is recommended to monitor infection dynamics.

## Discussion

The CFTR gene harbors more than 2,000 known variants, categorized into six classes based on their impact on protein function: Class I has no CFTR production, Class II results in CFTR protein misfolding and degradation. Class III involves gating defects (where the protein is produced but does not open properly), Class IV reduced conductance, Class V with lowered CFTR levels, and Class VI instability at the membrane (6).

To contextualize our patient's clinical course, we compared her outcomes with literature on infants homozygous for well-characterized Class I mutations (e.g, G542X, W1282X, R553X) (7, 8). Patients with these variants typically present before 6 months of age with meconium ileus (absent in our case), profound failure to thrive, and minimal weight recovery despite intervention. In contrast, our patient achieved weight catch-up to the 25th percentile by 18 months—a trajectory less severe than classic Class I profiles. This divergence may reflect the limitations of single-patient observations, differences in care intensity, or uncharacterized genetic modifiers. Thus, while c.1375_1383del aligns with Class I pathogenicity, definitive conclusions about its relative severity require larger cohort studies.

The c.1375_1383del variant is an in-frame deletion of nine nucleotides in exon 10 of the CFTR gene, resulting in the removal of three amino acids (Ser459-Gly461) within the first nucleotide-binding domain (NBD1). According to CFTR2, this variant is expected to result in little or no CFTR protein, likely due to impaired protein synthesis or instability (7). In-frame deletions like c.1375_1383del may arise from nonhomologous recombination in low-complexity genomic regions (9), a mechanism previously implicated in CFTR misfolding and degradation. While CFTR2 classifies this variant as Class I, experimental validation is needed to confirm its precise impact on protein function. Notably, the functional impact of c.1375_1383del remains experimentally uncharacterized. Structural studies are required to elucidate its precise biophysical consequences (4, 6).

This classification has critical therapeutic implications. While Class II mutations such as F508del have shown responsiveness to CFTR correctors like lumacaftor and tezacaftor, Class I mutations like c.1375_1383del—characterized by minimal or non-functional CFTR protein—are unresponsive to both correctors and ivacaftor monotherapy. This fundamental limitation highlights the need for alternative therapeutic strategies targeting protein restoration rather than modulation.

This case illustrates an early-onset CF phenotype with significant complications in a patient homozygous for c.1375_1383del, including recurrent pulmonary infections, failure to thrive, ARDS, and early bronchiectasis—all classical features of CF. Although Banjar et al. (4) previously reported this variant, no clinical phenotypes were described, making direct severity comparisons difficult. Our patient's complications—ARDS, failure to thrive, and early bronchiectasis—align with the clinical spectrum of Class I genotypes.

In regions with high consanguinity, such as Saudi Arabia, rare CFTR mutations like c.1375_1383del may be encountered more frequently. The absence of a national newborn screening program for CF likely contributed to the diagnostic delay observed here. Given that sweat chloride testing was unavailable at our center, whole-exome sequencing proved essential for diagnosis. This experience reinforces the urgent need for implementing universal newborn screening and integrating genomic diagnostics into pediatric respiratory care (10).

Looking ahead, investment in theratyping platforms such as patient-derived organoids (11, 12) will be critical for: (1) validating the functional consequences of rare variants like c.1375_1383del, and (2) guiding development of novel therapies for Class I mutations. Future studies should examine long-term outcomes of rare CF variants across diverse populations (13, 14) to optimize personalized management strategies.

Emerging therapeutic approaches for Class I CFTR mutations, such as c.1375_1383del, offer promise for future management. Investigational strategies, including mRNA therapy to deliver functional CFTR mRNA, gene therapy to introduce a correct CFTR gene, and read-through agents for certain Class I mutations with premature stop codons, are under development (15). These therapies are not yet clinically available for c.1375_1383del, but offer hope for personalized treatment of rare variants unresponsive to current CFTR modulators like lumacaftor or tezacaftor.

## Conclusion

The c.1375_1383del variant is a rare CFTR deletion with limited published data regarding its functional consequences and clinical outcomes. This case supports its classification as a CF-causing mutation, with a phenotype broadly consistent with other Class I genotypes. While early respiratory compromise was present, the patient's stabilization and catch-up growth suggest a clinical course within the expected spectrum. Long-term functional studies are warranted to clarify this variant's molecular behavior**.** This highlights the diagnostic value of genetic testing in high-risk populations and the importance of registering rare variants. Further functional studies are needed to clarify the molecular impact of this mutation and to guide potential future therapeutic strategies.

## Data Availability

Data from this case report cannot be shared publicly due to ethical and privacy concerns involving sensitive patient information. Publication complies with guardian consent and IRB approval (IRB-2024-01-576), ensuring anonymity and adherence to data protection laws. For inquiries about de-identified data, contact the author subject to IRB approval.
